# Mechanistic Insights into Potassium-Conferred Drought Stress Tolerance in Cultivated and Tibetan Wild Barley: Differential Osmoregulation, Nutrient Retention, Secondary Metabolism and Antioxidative Defense Capacity

**DOI:** 10.3390/ijms222313100

**Published:** 2021-12-03

**Authors:** Shafaque Sehar, Muhammad Faheem Adil, Muhammad Zeeshan, Paul Holford, Fangbin Cao, Feibo Wu, Yizhou Wang

**Affiliations:** 1 Department of Agronomy, Zijingang Campus, College of Agriculture and Biotechnology, Zhejiang University, Hangzhou 310058, China; 21516206@zju.edu.cn (S.S.); dradilfaheem@zju.edu.cn (M.F.A.); 11616102@zju.edu.cn (M.Z.); caofangbin@zju.edu.cn (F.C.); wufeibo@zju.edu.cn (F.W.); 2 Hawkesbury Campus, School of Science and Health, University of Western Sydney, Penrith, NSW 2751, Australia; p.holford@westernsydney.edu.au; 3 Jiangsu Co-Innovation Center for Modern Production Technology of Grain Crops, Yangzhou University, Yangzhou 225009, China; 4 Provincial Key Laboratory of Crop Germplasm, Zhejiang University, Hangzhou 310058, China

**Keywords:** drought stress, antioxidant enzymes, osmolytes, ATPase activity, secondary metabolism, drought responsive gene expression

## Abstract

Keeping the significance of potassium (K) nutrition in focus, this study explores the genotypic responses of two wild Tibetan barley genotypes (drought tolerant XZ5 and drought sensitive XZ54) and one drought tolerant barley *cv*. Tadmor, under the exposure of polyethylene glycol-induced drought stress. The results revealed that drought and K deprivation attenuated overall plant growth in all the tested genotypes; however, XZ5 was least affected due to its ability to retain K in its tissues which could be attributed to the smallest reductions of photosynthetic parameters, relative chlorophyll contents and the lowest Na^+^/K^+^ ratios in all treatments. Our results also indicate that higher H^+^/K^+^-ATPase activity (enhancement of 1.6 and 1.3-fold for shoot; 1.4 and 2.5-fold for root), higher shoot K^+^ (2 and 2.3-fold) and Ca^2+^ content (1.5 and 1.7-fold), better maintenance of turgor pressure by osmolyte accumulation and enhanced antioxidative performance to scavenge ROS, ultimately suppress lipid peroxidation (in shoots: 4% and 35%; in roots 4% and 20% less) and bestow higher tolerance to XZ5 against drought stress in comparison with Tadmor and XZ54, respectively. Conclusively, this study adds further evidence to support the concept that Tibetan wild barley genotypes that utilize K efficiently could serve as a valuable genetic resource for the provision of genes for improved K metabolism in addition to those for combating drought stress, thereby enabling the development of elite barley lines better tolerant of abiotic stresses.

## 1. Introduction

Due to global warming and subsequent climatic abnormalities, plants have evolved to live in environments where they are often exposed to a wide array of stresses such as drought, salinity, waterlogging and extreme temperatures [1,2]. Conspicuously, drought stress, in particular, causes multidimensional deleterious effects on plant growth, development and productivity. On a global scale, careful projections indicate that drought alone will affect more than 50% of the arable lands by the year 2050 [3,4]. To acclimatize water scarcity, plants go through various events at morphological (reduced biomass and nutrient uptake), physiological (reduced photosynthesis, altered transpiration and stomatal activity, noticeable reduction in cell growth), biochemical (accumulation of osmolytes, reduced carbohydrates metabolism and increased oxidative enzyme activity), and molecular level [5]. Despite a large number of studies, our understanding of the mechanisms of drought tolerance in barley is still not comprehensive due to the complexity of interactions involved in the physio-biochemical and molecular processes [6]. One of the most cost-effective solutions for sustainable production in water-limiting areas is genetic improvement for higher drought tolerance that could be achieved by understanding the underlying mechanism and identifying the associated genes. 

One of the main mechanism plants use to combat drought stress is the maintenance of normal cellular physiology through osmotic adjustment [1], which include the synthesis of new metabolites (such as callose, chitinase, phenols and flavonoids), and accumulation of osmolytes (such as proline, glycine and betaine), which interplay in protein solubilization and also share a pivotal role as reactive oxygen species (ROS) scavengers [7,8]. Although required for cell signaling, higher concentrations of ROS can instigate lipid peroxidation and degradation of vital proteins, lipids, and nucleic acids [8,9]. Thereby, plants have devised several enzymatic mechanisms including superoxide dismutase (SOD), catalase (CAT), and ascorbate peroxidase (APX), to persist against the oxidative damage caused by resultant ROS [10]. Leaf size reduction is another strategy adopted by plants that can directly influence the rate of transpiration; however, this reduction might lead to a decline in the photosynthetic activity [11]. In addition, many other biological processes and metabolic pathways involved in drought stress responses and tolerance have also been identified in gene expression studies. For example, using drought-tolerant and -susceptible accessions of barley (*Hordeum vulgare* L.) and wild barley (*Hordeum spontaneum* (K. Koch) Thell.), Guo et al. [12] identified 17 genes associated with drought tolerance that involved: carbon metabolism, the synthesis of glycine-betaine, scavenging of reactive-oxygen species, and stabilization of membranes and proteins.

Potassium (K) performs crucial functions in many physiological and biochemical activities during plant growth and development, including osmoregulation, maintenance of cytosolic pH, stabilization of protein synthesis, neutralization of negatively charged proteins, enzyme activation, photosynthesis, phloem loading and transport as well as the efficient use of other nutrients [13,14]. Drought stress interrupts K diffusion in soil towards the roots, thus restricting K absorption [15]. Hence, maintaining adequate cellular K is key to drought tolerance [16] and breeding better-adapted crop varieties is the most effective approach to this. Despite the modulation of drought tolerance by K [3,16], there is still a void in our understanding of whether K plays a role as a chemical signal between roots and leaves under drought, and whether higher K content is connected with better K retention in the leaf mesophyll. In particular, data related to genotypic differences in the tolerance of barley to combined drought and K deficiency stresses remain scarce. 

Barley is a major cereal crop that is mainly cultivated under rain-fed conditions and, as a result, can encounter drought during the grain-filling period [3,6]. Tibetan annual wild barley (*H. vulgare* L. ssp. *spontaneum* (K. Koch) Thell.) is a precious germplasm resource, and has a rich genetic diversity that may provide elite alleles for crop improvement in contrast to cultivated barley that has lost its robustness during the domestication process, making it hypersensitive to both abiotic and biotic stresses [17]. Our previous study revealed that Tibetan wild barley XZ5 is highly effective in K uptake and translocation, especially under drought conditions [16,18], but the morpho-biochemical and molecular response to drought remains unclear. Accordingly, the present study aimed to elucidate the physio-metabolic and molecular changes in response to K nutrition and drought stress tolerance in Tibetan wild barley XZ5 compared with drought sensitive wild barley XZ54 and drought tolerant *cv*. Tadmor which would help better understand the mechanisms of drought tolerance and provide an effective pathway for the exploration of drought tolerant genes in a barley crop. 

## 2. Results

### 2.1. Plant Growth, Chlorophyll and Photosynthetic Attributes

XZ5 exhibits excellent drought resistance in the presence of potassium. Compared with plants in the control treatment, significant decreases were observed in shoot heights (SH), root lengths (RL) and dry biomasses (SDW: shoot dry weight; RDW: root dry weight) in the –K+D treatment after 7 days (Table 1, Table S1). With K added to the drought treatment (+K+D), the effects of PEG-induced drought were alleviated to a greater extent in XZ5 (14%, 19% and 16% improvement for SH, RL and SDW, respectively), followed by Tadmor (a 21% improvement in RDW), when compared with –K+D treated plants. Moreover, XZ5 plants retained their root dry biomass under +K+D closer to +K (control). Although, no significant difference in shoot biomass was observed in XZ54 between +K+D and –K+D, a recovery rate of 6%, 10% and 14% was recorded for RDW, SH and RL, respectively. Under +K+D, relative chlorophyll contents (SPAD values), upon comparison with –K+D, had an ameliorative effect of 11%, 16% and 7% in XZ5, Tadmor and XZ54, respectively (Figure S1, Table S2). The Fv/Fm ratios of all genotypes were decreased by 2%, 5.6% and 9% under +K+D than control, whereas the provision of K helped the plants to reduce PSII damage (up to 15%, 11% and 16% in XZ5, Tadmor and XZ54, respectively), against –K+D. In terms of the photosynthetic parameters, *Pn*, *Gs*, *Ci* and *Tr*, both K deficiency treatments (–K and –K+D) significantly reduced all below control values (Figure S2, Table S2); for XZ5 and Tadmor, the addition of K had a pronounced alleviating effect on all the recorded parameters except for *Ci*.

### 2.2. Plants K, Ca, Mg and Na Concentrations

Shoots of all three genotypes subjected to +K+D showed a significant increase in Ca uptake compared to +K treated plants (Table 2, Table S3). The –K+D treatment also increased shoot Ca uptake in XZ5 but not in the other two genotypes. In the roots, the treatments had little effect on Ca concentrations. In the +K+D treatment, drought reduced K uptake in the shoots of Tadmor and XZ54, but had little effect in XZ5 compared to +K. In the roots of plants under +K+D treatments, drought reduced the K concentration in all tested genotypes, however, the extent of K concentration reduction was quite distinct under –K+D. Regarding concentrations of Mg in the shoots, the plants under –K treatment tended to have higher Mg concentrations than plants in the two +K treatments. Under –K treatments drought reduced Mg concentrations in the shoots of Tadmor and XZ54 but not in XZ5. In the roots, drought reduced Mg concentrations below levels in their non-droughted counterparts in XZ5 and XZ54 only with no significant difference under any treatment in Tadmor. For Na concentrations in the shoots, for XZ5 and Tadmor, and with the exception of Tadmor given the –K treatment, there were no significant differences in the concentration of this element among the treatments. For XZ54, shoot concentrations tended to be higher in plants given –K treatments. In addition, plants subjected to drought had higher Na concentrations than their non-droughted counterparts. In the roots, for all genotypes, the –K treatment more than doubled the concentrations of Na above those in the other treatments; however, there were no significant differences among the other treatments. With regard to the Na^+^/K^+^ ratios in the shoots, there was no effect of the treatments in XZ5. However, for Tadmor and XZ54, there was a trend for this ratio to be higher in droughted plants than their non-droughted counterparts and for the ratio to be lower in plants given +K treatments compared to those in –K treatments. In the roots, this ratio was lower in the plants given the +K treatments than their –K counterparts; however, there was no effect of drought treatments.

### 2.3. Antioxidative Enzyme Activities

**Figure 1:**
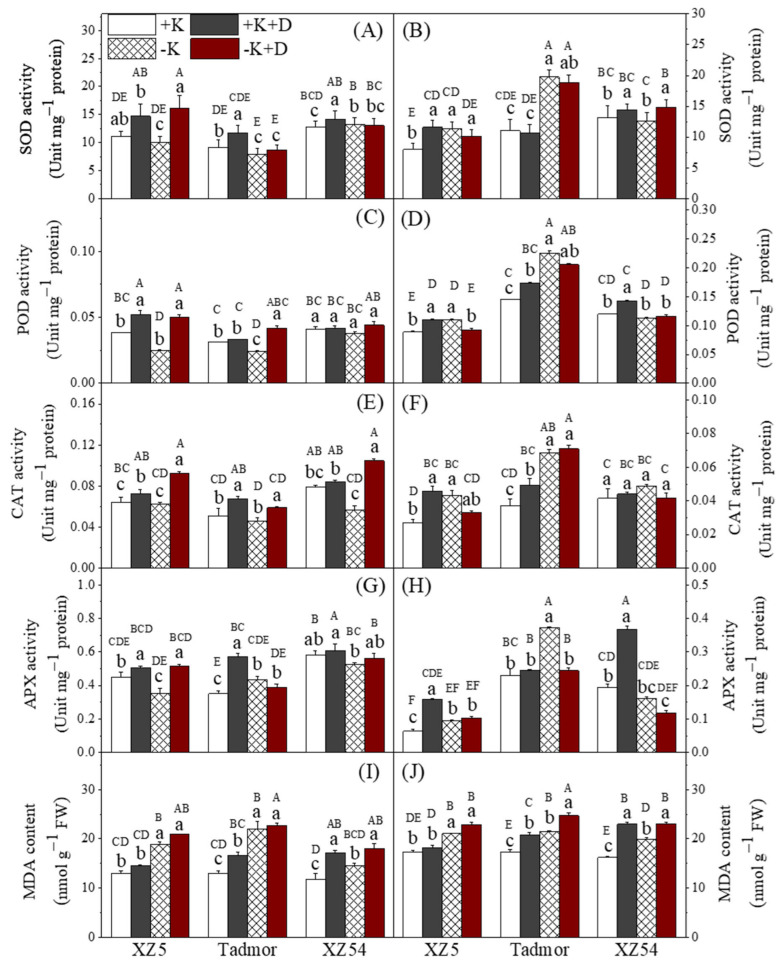
Effect of alone and combined stress of drought and K deprivation on antioxidant enzyme activities of SOD (**A**,**B**), POD (**C**,**D**), CAT (**E**,**F**), APX (**G**,**H**) and MDA contents (**I**,**J**) in leaves (left panel) and roots (right panel) of three barley genotypes. Different capital and small letter(s) indicate significant differences (*p* < 0.05) among the genotypes and among the 4 treatments within each genotype, respectively. +K (control), +K+D, –K, and –K+D, correspond to basic nutrition solution with normal K of 1 mM K (BNS), BNS + 20% polyethylene glycol 6000 (PEG), BNS without K (K deprivation), and BNS without K + PEG, respectively. Error bars represent standard deviations (*n* = 3).

Noticeable differences in antioxidant enzyme activity were seen in response to the K and drought treatments (Figure 1, Table S4). In the shoots, in general for all enzymes and for all genotypes, plants in the +K treatment (control) had the lowest activities. Two other trends were also found. Firstly, activities tended to be higher in plants given drought treatments in comparison to their non-droughted counterparts. Secondly, for SOD, APX and malondialdehyde (MDA), activities tended to be higher in –K and –K+D treatments than those where K was supplied. In the roots, there was little effect of the treatments on CAT activity in any of the genotypes. For the remaining enzymes, activities were higher in the +K+D treatment than the +K treatment. Comparing the –K+D and +K+D treatments, for XZ5, there were no differences between these treatments, for Tadmor, SOD, POD APX activities and MDA contents were lower in the –K+D treatment and MDA contents were higher, and for XZ54, only SOD activities and MDA contents were increased in the –K+D treatment. The effect of the treatments on the activities of the two ATPases was variable. For both enzymes, the trends in the activities tended to be similar in the roots and shoots of each genotype. For the Ca^2+^/Mg^2+^-ATPase in XZ5, activities of this enzyme were increased by both the drought and K deprivation treatments in both shoots and roots (Figure S3A,B). In the shoots of Tadmor, the activity of this enzyme was increased under –K and –K+D and under both drought and K deprivation in the roots. For XZ54, there was no effect of the treatments in either roots or shoots. H+-ATPase activity (Figure S3C,D) in the shoots and roots of XZ5 was also increased by +K+D and –K+D treatments. In the shoots of both Tadmor and XZ54, the –K treatment resulted in the lowest activities, with activities in the other treatments being similar. In the roots of Tadmor, the two drought treatments increased the activity of this enzyme. In XZ54, H^+^-ATPase activity was increased in the +K+D and –K treatments (Figure S3, Table S5).

### 2.4. Proline, Glycine Betaine, Soluble Sugars and Proteins Contents

**Figure 2:**
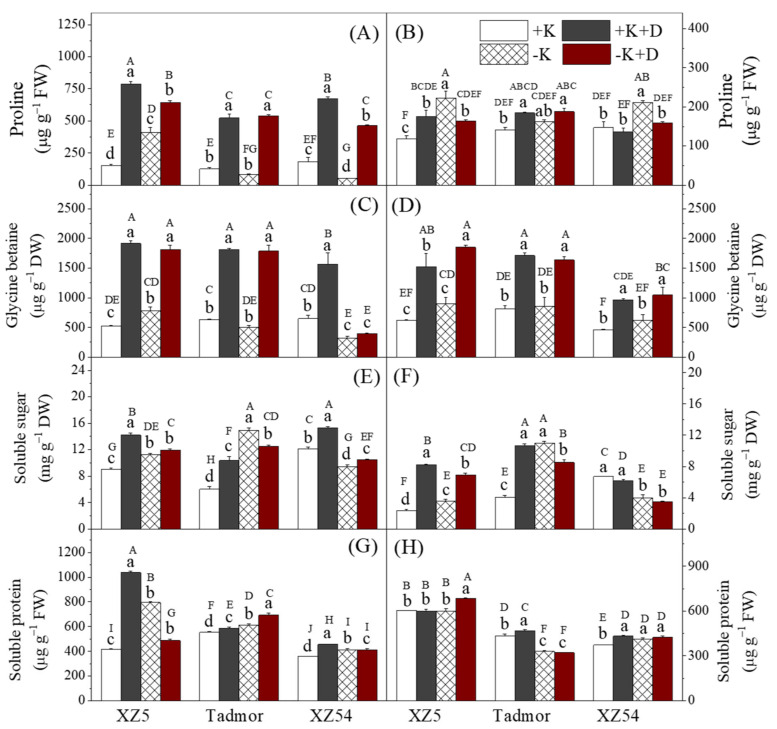
Effect of alone and combined stress of drought and K deprivation on proline, glycine betaine, soluble sugar and soluble protein contents in leaves (**A**,**C**,**E**,**G**) and roots (**B**,**D**,**F**,**H**) of three barley genotypes. Different capital and small letter(s) indicate significant differences (*p*<0.05) among the genotypes and among the 4 treatments within each genotype, respectively. +K, +K+D, –K, and –K+D, correspond to basic nutrition solution with normal K of 1 mM K (BNS), BNS + 20% polyethylene glycol 6000 (PEG), BNS without K (K deprivation), and BNS without K + PEG, respectively. Error bars represent standard deviations (*n* = 3).

In general, drought increased the concentrations of proline in the shoots and GB in shoots and roots of all genotypes (Figure 2, Table S6); for XZ5, the shoot concentrations of these compounds were also increased in the –K treatment compared to the +K control. Concentrations of proline in the roots of XZ5 and Tadmor were increased by both the drought and the –K treatments. For XZ54, only the –K treatment increased the concentration of this compound. With respect to both soluble sugars and proteins in the shoots, with the exception of soluble sugars in XZ54, both the drought and K deprivation treatments increased concentrations of these compounds above concentrations in the +K control; in XZ54, soluble sugar concentrations were increased in the +K+D treatment but reduced in the other two treatments. In the roots, the effect of the treatments on these two groups of compounds was variable among the genotypes. For soluble sugars, concentrations of these compounds were increased by the two drought treatments in XZ5, increased by all three treatments in Tadmor, and for XZ54, sugars were decreased by the two –K treatments. For concentrations of soluble proteins in the roots, the treatments had little effect with increases or decreases compared to the control being small.

### 2.5. Secondary Metabolism-Related Enzyme Activities

**Figure 3:**
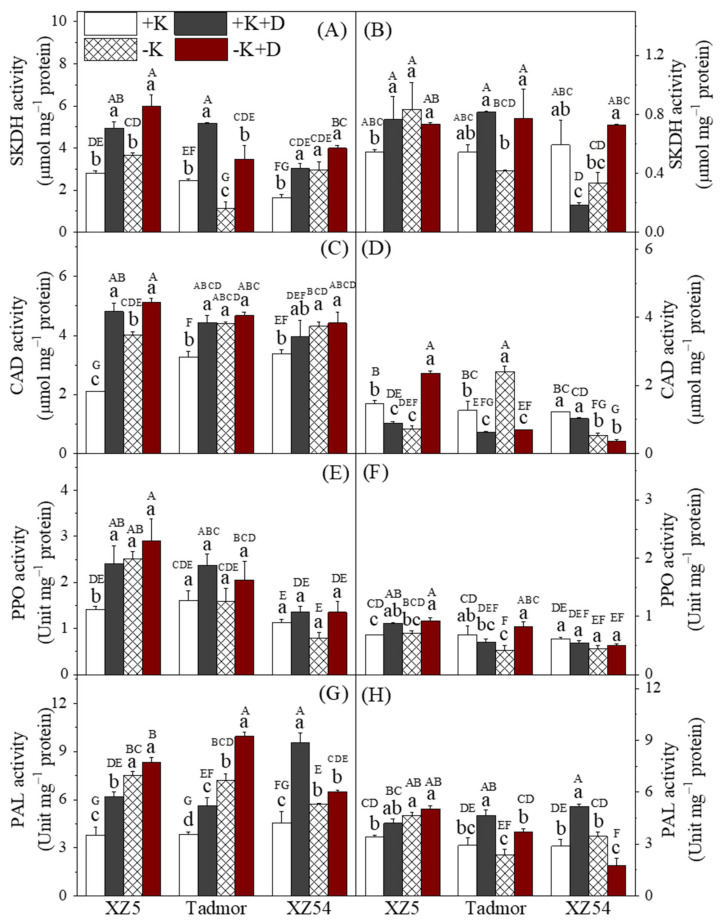
Effect of alone and combined stress of drought and K+ deprivation on secondary metabolism-related enzymes PAL, PPO, CAD and SKDH activities in leaves (**A**,**C**,**E**,**G**) and roots (**B**,**D**,**F**,**H**) of three barley genotypes. Different capital and small letter(s) indicate significant differences (*p* < 0.05) among the genotypes and among the 4 treatments within each genotype, respectively. +K, +K+D, –K, and –K+D, correspond to basic nutrition solution with normal K of 1 mM K (BNS), BNS + 20% Polyethylene glycol 6000 (PEG), BNS without K (K deprivation), and BNS without K + PEG, respectively. Error bars represent standard deviations (*n* = 3).

The activities of enzymes related to secondary metabolism in shoot and roots are presented in Figure 3 and Table S7. In the shoots, the two drought treatments increased the activities of all enzymes. Also, in general, the activities of these enzymes were also increased by the –K treatment. Activities of these enzymes in the roots were variable among genotypes, treatments and enzymes. For shikimate dehydrogenase (SKDH) in the roots of XZ5 and Tadmor, the drought treatments increased activities; the –K treatment also increased SKDH activity in XZ5, but reduced it in Tadmor. In XZ54, the +K+D treatment resulted in reduced SKDH activity. For cinnamyl alcohol dehydrogenase (CAD), in most incidences, the activities of this enzyme were reduced below control levels by the three treatments. For polyphenol peroxidase (PPO) in the roots, there were little effects of the treatments, and differences from the controls were small. Lastly, for phenylalanine ammonialyase (PAL), the drought treatments tended to increase activities in XZ5 and Tadmor; the –K treatment also resulted in increased activity in XZ5. For XZ54, PAL activity was increased above control levels in the +K+D treatment and reduced in the –K+D treatment.

### 2.6. Secondary Metabolites

**Figure 4:**
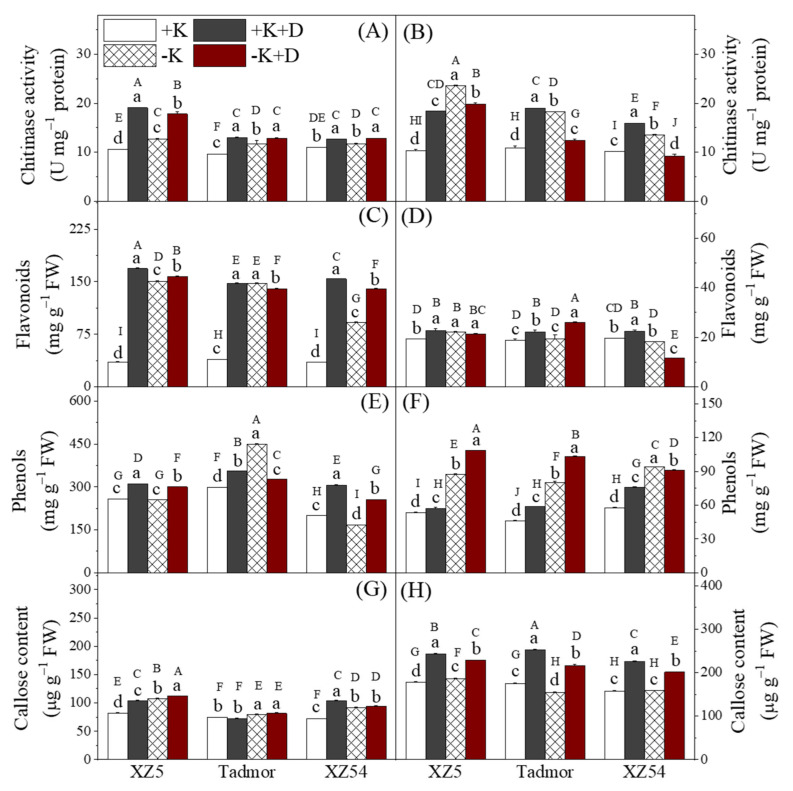
Effect of alone and combined stress of drought and K deprivation on secondary metabolites callose, phenol, flavonoids and chitinase activity in leaves (**A**,**C**,**E**,**G**) and roots (**B**,**D**,**F**,**H**) of three barley genotypes. Different capital and small letter(s) indicate significant differences (*p*<0.05) among the genotypes and among the 4 treatments within each genotype, respectively. +K, +K+D, –K, and –K+D, correspond to basic nutrition solution with normal K of 1 mM K (BNS), BNS + 20% polyethylene glycol 6000 (PEG), BNS without K (K deprivation), and BNS without K + PEG, respectively. Error bars represent standard deviations (*n* = 3).

The activity of chitinase in the shoots (Figure 4A) was increased in XZ5 and Tadmor by all treatments. For XZ54, the drought treatments also increased the activity of this enzyme in the shoots; however, the activity was not significantly different from the control in the –K treatment. In the roots, with the exception of plants of XZ54 given the –K+D treatment, chitinase activity was higher in all genotypes and treatments (Figure 4 B); for plants of XZ54 given the –K+D treatment, chitinase activity was lower than the controls. In the shoots, flavonoid contents were greatly increased by the drought and K deprivation treatments compared to the controls (Figure 4C). In the roots, flavonoid contents were lower than in the shoots and differences from the control were small (Figure 4D). The +K+D treatment increased the concentration of these compounds compared to the control in all three genotypes, the –K treatment only increased flavonoids in XZ5, and the –K+D treatment increased flavonoid contents in XZ5 and Tadmor, but reduced them in XZ54. Total phenolic contents in shoots were increased above control levels by the two drought treatments (Figure 4E). The –K treatment had variable effects on the three genotypes, having no effect on concentrations in XZ5, increasing them in Tadmor and reducing them in XZ54. In the roots (Figure 4F), both drought and K deprivation increased TP contents, with a possible interaction occurring between drought and K deprivation in XZ5 and Tadmor. Callose contents are presented in Figure 4 G and H. In the shoots, the two K deprivation treatments (–K and –K+D) increased callose concentrations in all three genotypes; the +K+D treatment increased concentrations in XZ5 and XZ54 but not in Tadmor. In the roots, callose concentrations were higher than the shoots. The two drought treatments increased the contents of this compound in all three genotypes; the –K treatment had little effect on callose concentrations (Figure 4, Table S8).

### 2.7. Gene Expression Associated with ROS Scavenging and Secondary Metabolism

**Figure 5:**
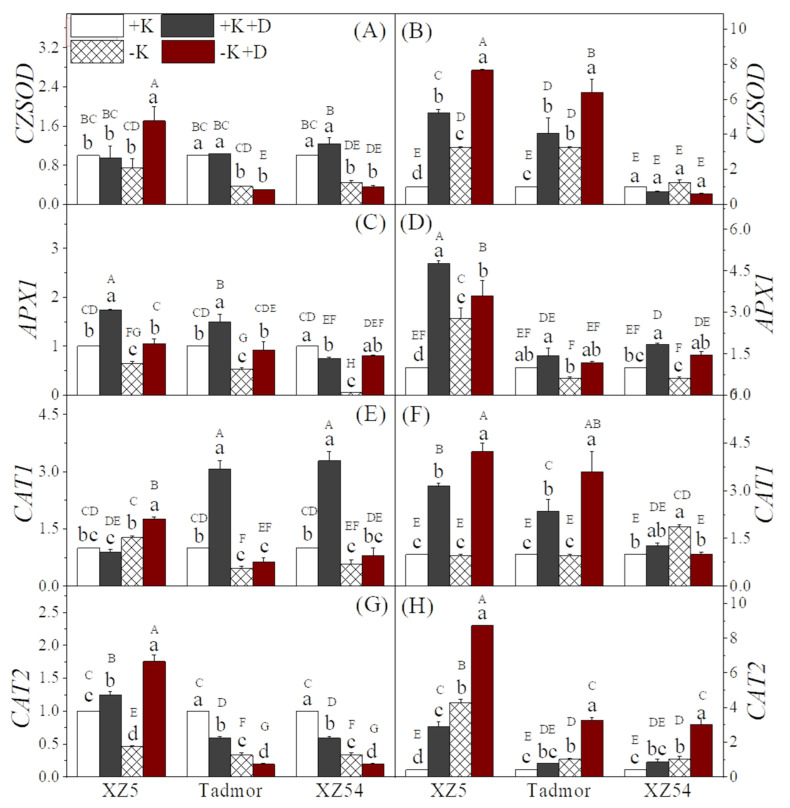
Antioxidant enzyme related gene expression analyzed by qRT-PCR in Tibetan wild barley XZ5 and XZ54, and cv. Tadmor. Barley plants were exposed to alone and combined stresses of drought and K+ deprivation on CZSOD, APX1, CAT1 and CAT2 gene expression study in leaves (**A**,**C**,**E**,**G**) and roots (**B**,**D**,**F**,**H**) of three barley genotypes, XZ5, Tadmor and XZ54. Different capital and small letter(s) indicate significant differences (*p* < 0.05) among the genotypes and among the 4 treatments within each genotype, respectively. Error bars represent standard deviations (*n* = 4). +K, +K+D, –K, and –K+D, correspond to basic nutrition solution with normal K of 1 mM K (BNS), BNS + 20% Polyethylene glycol 6000 (PEG), BNS without K (K deprivation), and BNS without K + PEG, respectively.

To understand the changes at the molecular level caused by drought and K deprivation, alone or in combination, on antioxidant enzyme-associated genes, the transcript levels of CZSOD, APX1, CAT1 and CAT2 genes were investigated (Figure 5, Table S9). For CZSOD, in XZ5, only the –K+D treatment showed a difference from the control (a 1.7-fold increase); for the other two genotypes the two –K treatments reduced expression, while in roots, for XZ5 and Tadmor, both the –K and drought treatments increased expression; there were no significant differences from the control in XZ54 (Figure 5A,B). In the shoots, for XZ5 and Tadmor, the expression of APX1 was increased by the two drought treatments (Figure 5C), whereas the shoots of XZ54 exhibited a contrasting trend. In the roots, both drought and –K treatments increased expression of APX1 (Figure 5 D) in XZ5 whereas, for the other two genotypes, only the drought treatments displayed increases in expression above the controls. The shoot and root CAT1 transcript levels of the three genotypes are presented in Figure 5E,F. In the shoots, for XZ5, only the –K+D treatment increased transcript levels; the other treatments had no effect. For the other two genotypes, the +K+D treatment greatly increased expression; the two K deprivation treatments tended to reduce transcript levels. In the roots, the two drought treatments greatly increased expression of CAT1 in XZ5 and Tadmor; in XZ54, the –K and +K+D treatments increased expression. For transcript levels of CAT2 in the shoots, for XZ5, the two drought treatments increased expression whereas the –K treatment reduced it. For Tadmor and XZ54, expression of CAT2 was reduced by both the drought and K deprivation treatments. In the roots, there was a trend for both drought and K deprivation to increase transcript levels (Figure 5H).

**Figure 6:**
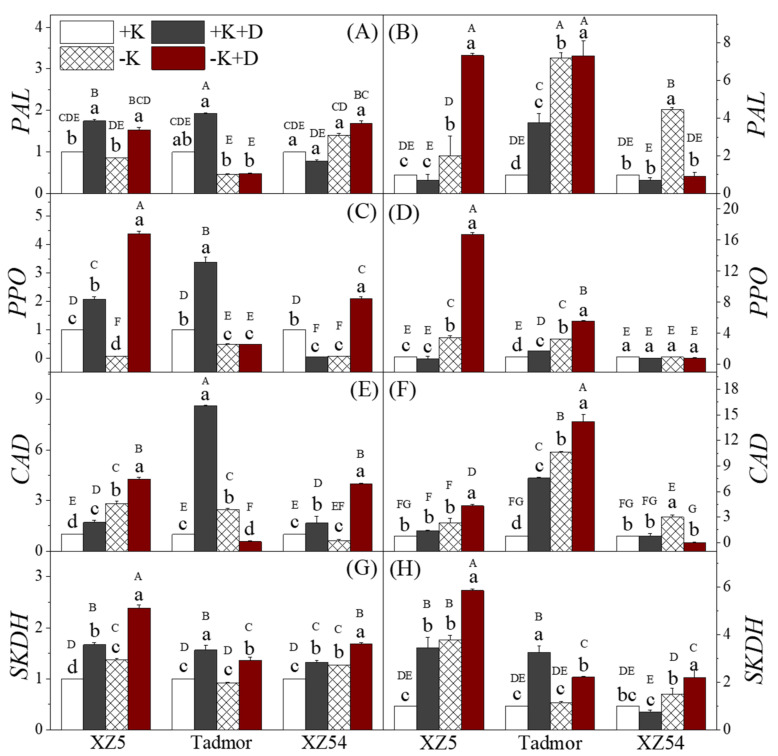
Secondary metabolism related gene expression analyzed by qRT-PCR in Tibetan wild barley XZ5 and XZ54, and cv. Tadmor. Barley plants were exposed to alone and combined stresses of drought and K deprivation, PAL, PPO, CAD and SKDH gene expression were studied in leaves (**A**,**C**,**E**,**G**) and roots (**B**,**D**,**F**,**H**) of three barley genotypes. Different capital and small letter(s) indicate significant differences (*p*<0.05) among the genotypes and among the 4 treatments within each genotype, respectively. Error bars represent standard deviations (*n* = 4). +K, +K+D, –K, and –K+D, correspond to basic nutrition solution with normal K of 1 mM K (BNS), BNS + 20% Polyethylene glycol 6000 (PEG), BNS without K (K deprivation), and BNS without K+ PEG, respectively.

To gain a better understanding at the molecular level of changes in secondary metabolism, the transcript levels of the PAL, PPO, CAD and SKDH genes were studied (Figure 6, Table S10). In general, for all enzymes, expression varied with both treatment and genotype and their transcript levels were in agreement with physiological data. Shoots of XZ5 displayed increased expression of PAL under both drought treatments (Figure 6A), while in Tadmor, the two K deprivation treatments reduced its transcript levels. In the roots (Figure 6B) of the two drought tolerant genotypes, the expression of PAL was increased by the two K deprivation treatments, whereas in XZ54, expression was only enhanced under –K. A significant increase in PPO expression in XZ5 shoots was observed in the two drought treatments (Figure 6C). In Tadmor, only +K+D increased the expression of this enzyme, and the other two treatments reduced expression. In XZ54, expression was increased in the –K+D treatment but decreased in the other two. Moreover, the two K deprivation treatments increased expression of PPO in XZ5 and Tadmor roots, but there were no significant differences among the treatments in XZ54 (Figure 6D). The expression of CAD in the shoots was increased by the two drought treatments in XZ5 and XZ54 (Figure 6 E), but only by the +K+D treatment in Tadmor. In the roots, for the two drought tolerant genotypes, there was a trend for all treatments to increase the expression of this gene; however, these effects were greater in Tadmor (Figure 6F). Figure 6 G,H present the changes in expression of SKDH. The expression of this gene was increased by the two drought treatments in all genotypes; the –K treatment also increased expression in shoots of XZ5 and XZ54, whereas the roots of two drought tolerant genotypes showed increased expression of this gene under both drought treatments. Additionally, XZ5 roots exhibited increased levels of transcripts under –K. In XZ54, only the +K+D treatment increased SKDH expression.

## 3. Discussion

Tibetan wild barley constitutes an important genetic resource and contributes to pastoral production particularly in semi-arid regions because of its drought tolerance. In a previous study, Zhao et al. [16] reported that the Tibetan wild-barley genotype, XZ5, grows well under drought conditions; this could also be observed in our study. The severity of drought stress symptoms (reduction in biomass, shoot heights, root lengths, wilting and the appearance of chlorotic patches) were less in XZ5 compared to Tadmor and indicate the potential of wild barley as a source for drought tolerant alleles that can be used in the breeding of cultivated barley. In addition, XZ54 showed greater relative reductions in growth due to the K deprivation treatments suggesting that drought tolerance is at least in part associated with the homeostasis of this element (Table 1). Drought stress is well documented as reducing photosynthetic characteristics, shortening the duration of photosynthesis and promoting leaf senescence. A decrease in *Pn* limits expansion of the assimilation area of vegetative organs, and a notable reduction has been demonstrated in *Pn* during several investigations of drought stress in different plant species [19] as was found in our results. A large reduction in *Gs* and *Tr* is a consequence of stomatal closure in response to drought and is an important stress avoidance mechanism in plants [20]; reductions in these parameters were also found in our study (Figure S2). A greater decrease in Fv/Fm was observed in XZ54 in the two drought stress treatments as compared to XZ5 and Tadmor, which suggests a possible inhibition of PSII photochemistry in XZ54 (Figure S1). A higher protective capacity for PSII is an important tolerance mechanism for barley exposed to drought [12]. K can play a role in rescuing photosynthetic activities [13] and in this study, the two K deprivation treatments reduced certain photosynthetic parameters compared to plants given sufficient K.

Nutrient acquisition by roots and their transport to shoots is significantly affected by drought stress which restricts K diffusion in the soil towards the roots, thereby limiting K absorption [13,17]. In our study, there were significant genotypic differences in shoot Na^+^ and K^+^ concentrations in response to drought stress and K deprivation in the barley genotypes (Table 2). Potassium, being highly mobile, is easily translocated at both the cellular and plant levels and could translocate from older to younger leaves under K deficiency in genotypes that are tolerant of low K concentrations [21]. Our results indicate that tolerance to drought stress in XZ5 was associated with higher K uptake and translocation and a lower Na^+^/K^+^ ratio (Table 2). For XZ54 and Tadmor, their decreased photosynthetic capacity could also be attributed to an increased leaf Na^+^/K^+^ ratio, as has been shown by James *et al*. [22]. The genotypes showed clear differences in their abilities to take up K with the concentrations in shoots being in the same order as their drought tolerance. This suggests that XZ5 is a good source of genes for K uptake and also supports the contention of a link between K uptake and drought tolerance. Another clear trend was the enhanced concentration of Ca^2+^ in the shoots of all genotypes given the drought stress treatments, particularly in XZ5. This suggests a role for Ca^2+^ in drought stress signaling in XZ5, and accords with a previous study conducted by Tuteja and Mahajan [23], showing that cellular Ca^2+^ regulates gene expression and is also crucial for plant defense against various stresses. 

Studies on the interaction between drought and magnesium are relatively rare but a few have found reductions in leaf Mg contents due to drought [24]. In this study, drought per se did not affect leaf Mg concentrations. However, in the shoots, of Tadmor and XZ54, the combined effect of the –K and +D treatments reduced Mg concentrations, which is a reflection of greater interaction between these two treatments. In the roots, drought reduced Mg concentrations in XZ5 and XZ54; a similar trend was seen in Tadmor. Drought increases ROS production by chloroplasts, peroxisomes and mitochondria and this could be further enhanced in K-deficient plants [8,25]. K deficiency induces ABA accumulation that in turn induces the activity of nicotinamide adenine dinucleotide phosphate (NADPH)-dependent oxidases and the formation of NADPH-dependent ROS [11]. To counteract the resulting oxidative damage, plants have evolved a complex mechanism to induce antioxidative enzymes, including SOD, POD, APX and CAT [5,10], and increased activities of these enzymes in response to drought were found in all genotypes in this study (Figure 1). K deprivation also increased the activities of these antioxidant enzymes with the greatest effect in XZ5 and the least in XZ54; again, suggesting that adequate K nutrition may play a role in mediating drought tolerance. PPOs are also associated with a plant’s response to biotic and abiotic stresses including the response to water deficit catalyzing the reduction of H_2_O_2_ [25]. In our study, we also found a significant induction of PPO activity in the leaves of XZ5 and Tadmor under stress, the drought tolerant genotypes, but no changes in XZ54 (Figure 3). 

Genes encoding the antioxidant enzymes are differentially influenced by abiotic stresses in barley roots and leaves [1,10]. Drought increased expression of all four genes in the roots of XZ5, and CZSOD and CAT1 in Tadmor, suggesting that these drought-tolerant genotypes are better able to increase gene expression in response to drought stress (Figure 5). In addition to the enzymes mentioned above, plants contain non-enzymic means of scavenging ROS. Flavonoids arising from the shikimate pathway are associated with defense mechanisms [26], and are able to scavenge ROS. Flavonoid contents were increased by drought and K deprivation in the shoots of all three genotypes; however, there was little difference among them, neither were there marked differences among the genotypes and treatments in the roots. In addition to flavonoids, other phenolic compounds also play a role in the alleviation of oxidative stress and the detoxification of ROS [15]. Our study showed an increase in phenolic contents in the leaves and roots in response to drought with XZ5 and Tadmor having higher levels than XZ54 in the shoots (Figure 4). Most natural phenolic compounds in plants are derived from trans-cinnamic acid due to the action of PAL and correlations have been found between PAL activity, accumulation of phenolics and drought tolerance [27]. PAL activity was elevated in the leaves in response to both the drought and K deprivation treatments with, in general, XZ5 and Tadmor showing the greatest elevations; PAL activity in the roots of XZ5 tended to be higher than the other two genotypes, particularly in response to the drought treatments (Figure 3). Despite these changes in PAL activity, no obvious association with phenolic contents was found. MDA contents are often used as an indicator of the extent of lipid peroxidation resulting from oxidative stress [9]; lower MDA contents indicate reduced lipid peroxidation. In our study, the combined effects of drought and –K treatments are reflected by the difference in the levels of lipid peroxidation in both analyzed tissues (Figure 1).

Changes in the activities of Ca^2+^-Mg^2+^-ATPase in chloroplasts are crucial for plant functioning under stress and have reportedly been involved in drought tolerance in sweet potatoes [28]. The current study showed that –K+D treatment increased the activity of this enzyme in the roots of both drought-tolerant genotypes as well as increased the activity, particularly in the leaves of XZ5 (Figure S3). H^+^-ATPase is associated with a broad range of physiological processes including cell wall acidification, cytoplasmic pH regulation, and the provision of proton motive force for secondary active transport and K is absorbed by means of proton-mediated transport systems [29]. The activity of this enzyme being greatest in the leaves and roots of XZ5 could explain both its higher K uptake and drought tolerance of the three genotypes tested. Increased accumulation of compatible solutes has been indicated as an adaptive response to abiotic stress [8,30]. Proline is a well-known osmoprotectant in plants, and accumulates under saline conditions and functions as a protein stabilizer, a hydroxyl radical scavenger, a source of carbon and nitrogen and a cell membrane stabilizer [7]. In the present study, proline was higher in the roots of XZ5 and Tadmor than those of XZ54 subjected to +K+D and –K+D treatments potentially improving the hydration status of XZ5 and Tadmor. In addition, concentrations of soluble sugars were reduced by the two treatments in the roots of XZ54 but increased in the drought-tolerant genotypes (i.e., XZ5 and Tadmor). Lower osmotic potentials could increase the turgor pressure leading to higher water potentials in XZ5 and Tadmor than in XZ54, which also indicate they have higher water-holding capacities. GB has also been shown to have a beneficial role in plant species under abiotic stress, and accumulation of GB can improve their capacity for osmotic adjustment under drought stress [31] as was found in this study and accumulation was higher in the two drought tolerant genotypes than in XZ54. Soluble protein contents increased in tissues under all stresses but were higher in the two drought tolerant types. In the current study, protein contents were most increased in XZ5 and Tadmor suggesting a role in drought tolerance (Figure 2).

Enhanced CAD activity results in an increase in the synthesis of cinnamyl alcohols and is a specific marker of lignification. Drought treatments have been shown to increase CAD expression in *Linum usitatissimum* L. [32] and *Cucumis melo* L. [33], and these authors suggest that enhanced lignification improves the mechanical strength of root cells and helps alleviate osmotic stresses. In our experiment, CAD activity was induced by both drought and K deprivation in the shoots, but differences between genotypes were small; activities in the roots were low. The activity of SKDH, an enzyme involved in the production of aromatic amino acids, precursors of lignin, was also increased by the drought and the K deprivation treatments (Figure 6). This was particularly noticeable in XZ5 and Tadmor, and this enzyme may contribute to drought tolerance in general and to the better growth of the two tolerant genotypes. In addition to a role in plant development, chitinases have a participation in general stress response. Accordingly, a greater tolerance to drought was found in transgenic plants expressing chitinase genes [34]. In this study, XZ5 showed the greatest increases in chitinase activity in the shoots and roots in response to the drought treatments, suggesting that tolerance of drought in this genotype may be mediated by chitinase. Callose takes part in several important processes associated with plant development and is involved in responses to multiple biotic and abiotic factors [35]. Our results showed that both drought and K deprivation increased callose contents in the shoots with increases being greater in the wild barley genotypes; the drought treatments also increased callose contents in the roots of all genotypes but there was little difference among the genotypes. 

## 4. Materials and Methods

### 4.1. Plant Materials, Growth Conditions and Physiological Parameters

Greenhouse hydroponic experiments were conducted employing two Tibetan wild barley genotypes XZ5 (drought-tolerant) and XZ54 (drought-sensitive) (*Hordeum vulgare* L. ssp. *spontaneum*) screened and identified in our previous study [16], and a drought-tolerant *cv*. Tadmor. Seeds were surface sterilized with 2% H_2_O_2_ for 30 min, rinsed thoroughly with distilled water, and then germinated on sterilized moist quartz sand in germination trays in a growth chamber (22/18 °C day/night and 80% relative humidity (RH)). Uniform, healthy, 7-day-old seedlings were transplanted into 5.5 L containers filled with 5 L basal nutrient solution (BNS). The composition of BNS was as follows (mg L^−1^): KCl 74.5, Ca(NO_3_)2.4H2O 236, MgSO_4_.7H_2_O 98.4, NH_4_H_2_PO4 23, H_3_BO_3_ 0.185, MnCl_2_.4H_2_O 0.099, (NH_4_)_6_Mo_7_O_24_ 1.236, ZnSO_4_.7H_2_O 0.115, CuSO_4_.5H_2_O 0.05, Fe(III)-EDTA 8.42 [36]. The pH of the BNS was adjusted to 5.8 ± 0.1 with NaOH or HCl. The solution was continuously aerated with pumps and renewed every 4 days. After 7 days of acclimatization, seedlings were deprived of K (excluding KCl) for 5 days and then each genotype was divided into four different treatments: seedlings grown in BNS with normal K nutrition (1 mM) (+K, control); BNS + 20% PEG 6000 (+K+D); BNS without K (–K); and –K + 20% PEG 6000 (–K+D). The experiment was laid out as a completely randomized design with five replicates (in total 60 pots; each having 14 seedlings). After 7 days of PEG treatment, plants were sampled for the measurement of root length, shoot height and fresh weight. Fresh root and leaf samples were immediately frozen in liquid nitrogen and stored at −80 °C for further analyses. The remainder was dried for 3 h at 105 °C and then for another 24 h at 80 ± 1.5 °C to a constant dry weight for the measurement of dry biomass and elemental composition.

### 4.2. Chlorophyll and Photosynthetic Attributes

The second fully-expanded leaves (three plants per pot) of 26 days old plants were selected to measure relative chlorophyll contents using a chlorophyll meter (SPAD-502, Minolta; [37]). Chlorophyll fluorescence analysis was undertaken using an imaging pulse amplitude modulation (PAM) device (IMAG-MAXI; Heinz Walz, Effeltrich, Germany). After 20 min of dark adaption, leaves were illuminated with a saturating light pulse with a frequency of 0.05 Hz lasting 260 s. The maximal photochemical efficiency of PSII (Fv/Fm) was calculated according to [38] using Imaging Win software (Heinz Walz GmbH, Effeltrich, Germany). Photosynthetic parameters including net photosynthetic rate (*Pn*), stomatal conductance (*Gs*), intercellular CO_2_ concentration (*Ci*) and transpiration rate (*Tr*) were determined using an infrared analyzer (LI-6400 System; Li-COR, Lincoln, NE, USA). All measurements were performed on a sunny day between 10:00 a.m. and 1:00 p.m., with an air temperature of 23–25 °C, a RH between 50–70%, a CO_2_ concentration of 400 µmol mol^−1^, and a photosynthetic photon flux density (PPFD) of 1000 mol m^2^ s^−1^. 

### 4.3. Measurement of Elemental Concentration

Dried shoot and root samples (0.1 g) were ashed at 550 °C for 12 h. The ash was digested with 5 mL of 30% HNO3 and then diluted using deionized water. The concentrations of K, calcium (Ca), magnesium (Mg) and sodium (Na) were determined according to [39], using a flame atomic absorption spectrometer (Shimadzu, Kyoto, Japan).

### 4.4. Determination of Antioxidant Enzyme Activities and Malondialdehyde Contents

Plants (26 days old) were harvested and immediately kept on ice, then separated into roots and shoots. Two plants from each replicate were pooled together then divided into three parts, and out of each part, 0.5 g was homogenized in 10 mL of cold 50 mM phosphate buffer solution (PBS; pH 7.0) in an ice bath by grinding using a mortar and pestle. The homogenate was centrifuged at 4 °C for 20 min at 12,000 rpm. The activities of SOD (EC 1.15.1.1), CAT (EC 1.11.1.6) and POD (EC 1.11.1.7) were assayed according to [36], while APX (EC 1.11.1.11) activity was determined according to [25]. Levels of lipid peroxidation were expressed as the MDA contents following the methods of [40], where fresh plant material (0.1 g) from each treatment was homogenized in 2 mL of pre-cooled PBS. A reaction solution comprising 5% (*w/v*) trichloroacetic acid and thiobarbituric acid was added to the supernatants obtained after centrifugation. The absorbance of the supernatant was measured spectrophotometrically at 532 nm and corrected for turbidity using the absorbance at 600 nm. ATPase activities were determined by quantitating Pi released from ATP and pyrophosphate using an activity assay kit (Jiancheng Bioengineering Institutes, ,Nanjing, China), and the procedure described by manufacturers was followed.

### 4.5. Estimation of Osmoprotectants

The sulfosalicylic acid method of [41] was employed to determine proline concentrations. Briefly, samples (0.1 g) after homogenization in 5 mL sulfosalicylic acid (3%) were mixed with 1.25% ninhydrin in glacial acetic acid at a ratio of 1:2, and after boiling at 100 °C for 30 min was then directly used for spectrophotometric measurement at 508 nm. Standard curve with different proline concentrations was used for proline quantification. Determination of glycine betaine (GB) was undertaken using 0.5 g dried and powdered tissue samples, extracted in ddH_2_O for 24 h at 25 °C, and mechanically shaken. After filtration, 0.5 mL of extract was mixed with 1 mL of 2 N HCl solution. Then 0.1 mL of cold potassium tri-iodide solution (7.5 g iodine + 10 g KI in 100 mL of 1 N HCl) was added into it and shaken in an ice bath for 90 min and then 2 mL of chilled water was poured in. Finally, 10 mL of 1,2-dichloroethane (chilled at −10 °C) was added. From the two layers formed after 1-2 min, the upper layer was discarded and the optical density of the lower layer was recorded at 365 nm [42]. Standard curve developed with different concentrations of GB was used to estimate sample GB contents. Total soluble sugars were estimated using anthrone reagent [43] using glucose as a standard, samples (0.5 g) were extracted 3 times with ddH_2_O (5 mL each time) and 1 time with absolute ethanol for 10 min at 95 °C. Then the blue-green solution was measured for its optical density at 630 nm. Soluble protein concentrations were measured by a standard assay [44], using bovine serum albumin as a standard (Bio-Rad, Hercules, CA, USA), where sample amounting to 0.5 g after homogenization in 5 mL TRIS-HCl buffer (pH 7.4) was centrifuged at 12,000× g and filtered. Approximately, 0.02 mL of supernatant was utilized for the reaction which involved 2.5 mL of reagent (100 mg G250 + 47.5 mL absolute ethanol + 100 mL H_3_PO_4_). Readings were taken at 595 nm after 5 min reaction time.

### 4.6. Assessment of Phenol, Flavonoid, Callose Content and Chitinase Activity

Total phenolic contents (TP) were determined using 0.2 g frozen samples ground in 2 mL of 80% methanol and sand using a pestle and mortar in an ice bath followed by ultrasonication for 30 min. The reaction system was as follows: 150 μL Folin–Ciocalteu reagent (Sigma-Aldrich, Darmstadt, Germany), 200 μL supernatant and 2 mL 2% Na_2_CO_3_. Gallic acid was used to prepare a standard curve and absorbance was measured immediately at 735 nm [45]. Flavonoid contents were determined according to [46] using 0.2 g of frozen samples that were homogenized with 2 mL 1% HCl in methanol and sonicated as above. After centrifugation, 300 μL of supernatant, 300 μL 5% NaNO_2_, and 300 μL 10% AlCl_3_ were added, followed by 2 mL of 1 M NaOH. The absorbance was measured at 510 nm. For the quantitative measurements of callose contents, plant samples were ground and heated at 80 °C with 1 M NaOH. The homogenates were centrifuged at 1000× g for 10 min, then the supernatants were mixed with aniline blue solution (40 vol of 0.1% aniline blue in water, 21 vol of 1 M HCl, and 59 vol of 1 M glycine/NaOH buffer, pH 9.5) and their fluorescence measured using a spectrofluorimeter (Jobin-Yvon/SPEX) as described by [47]. The plant chitinase activity was determined using an ELISA kit (Sigma-Aldrich, Darmstadt, Germany), and expressed as picomoles of 4-methylumbelliferone liberated per min and per milligram of protein. 

### 4.7. Secondary Metabolism-Related Enzyme Activities

PAL activity was measured using 0.2 g homogenized plant samples as described by Ruiz et al. [48] and the absorbance recorded at 290 nm. CAD activity was measured following the oxidation of hydroxycinnamyl alcohol at 30 °C according to [49]. Absorption was measured at 400 nm with an interval of 20 s and an absorption coefficient of 21 mM cm^−1^. PPO activity was determined according to [48]. Shikimate dehydrogenase (SKDH) was determined according to [50]; absorption was measured at 340 nm with an interval of 20 s and an absorption coefficient of 6.22 mM cm^−1^. Protein contents were quantified according to [44] using bovine serum albumin as a standard. The spectrophotometric analyses were conducted in 96-well Corning® Costar® 96-Well Cell culture plates (Sigma-Aldrich, Darmstadt, Germany).

### 4.8. Total RNA Extraction, cDNA Synthesis and qRT-PCR Assay

Total RNA was extracted from 100 mg of leaf tissue using the Takara MiniBEST Plant RNA Extraction Kit (Takara, Tokyo, Japan) according to the manufacturer’s instructions. RNA concentrations were measured using a Titertek-Berthold nanospectrometer (Pforzheim, Germany), and for RNA quality assay, the samples were electrophoresed in a 1% agarose gel using 1 × TAE as the running buffer. A Prime script RT reagent Kit (Takara, Tokyo, Japan) was used for cDNA synthesis. The cDNA samples were assayed by quantitative real time PCR (qRT-PCR) in a Light Cycler^®^ 480 II Real-Time PCR System (Roche, Basel, Switzerland) using the SYBR^®^ Premix Ex Taq™ II (Takara, Tokyo, Japan). The primers used for the amplification of target cDNAs were designed using the barley genome available at the National Center for Biotechnology Information (https://www.ncbi.nlm.nih.gov/pubmed, accessed on 7 September 2020). The software provided with the PCR system was used to calculate threshold cycle values, and quantification of mRNA levels was performed according to the method of [51]. The *HvActin* gene (accession number AY145451) was amplified with the primer pair, 5′TTCTCGACTCTGGTGATGGTGT3′and 5′CAAGCTTCTCCTTGATGTCCCT3′, as an internal control for the relative amount of RNA. The specific primers used in this experiment are presented in Table S11.

### 4.9. Statistical Analysis

All data are presented as the mean values. Analysis of variance (ANOVA) was performed to determine statistical differences among the treatments. The significance level for the difference between Tibetan wild and cultivated barley genotypes, and among the control and K and D treatments were determined using LSD tests at (*p* < 0.05) via the Data Processing System (DPS) Software Package [52]. Graphical representations were prepared using Origin Pro version 2019b (Origin Lab Corporation, Wellesley Hills, Wellesley, MA, USA). 

## 5. Conclusions

A comparative tolerance to the combined effect of drought and K+ deficiency has been displayed by the Tibetan wild barley XZ5, and to a certain extent cv. Tadmor, relative to the drought susceptible genotype XZ54. However, improved K+ nutrition remains pivotal for promoting plant growth and mitigating the harsh effects of drought stress. Observations led to a conclusion that tolerant varieties tend to make the most of available K+ by its quick translocation to the shoot which improves overall water use efficiency. Stress tolerance is also mediated by multiple traits including maintenance of the photosynthetic apparatus, reduction in the effects of ROS, increased Ca accumulation in shoots to trigger stomatal closure, and production of compatible solutes suggesting that these traits may also mediate tolerance to a wide spectrum of abiotic stresses. Thus, our results add further evidence to support the concept that Tibetan wild barley is a valuable resource of genes for improved K metabolism in addition to those for combating drought stress. The exploitation of these genes will enable the development of elite barley lines better tolerant to abiotic stresses.

## Figures and Tables

**Table 1:** Effect of K nutrition on biomass, shoot height and root length of three barley genotypes in response to drought stress.

Genotype	Treatment	Shoot DW (µg plant^–1^)	Root DW (µg plant^–1^)	Shoot Height (cm)	Root Length (cm)
XZ5	+K	143.4 ± 8.94 ABCa	53 ± 0.86 ABa	27.0 ± 0.41 Ca	23.5 ± 1.21 Ba
	+K+D	139.0 ± 11.35 ABCa	51.09 ± 1.03 Ba	26.2 ± 1.06 Da	23.5 ± 1.30 Ba
	–K	123.8 ± 9.79 CDb	49.97 ± 1.70 BCab	24.9 ± 0.55 CDab	23.3 ± 0.81 Ba
	–K+D	119 ± 9.35 CDb	45 ± 2.35 Cb	23.0 ± 0.19 Eb	19.7 ± 1.09 DEb
Tadmor	+K	150 ± 3.88 ABa	43 ± 2.76 CDa	22.6 ± 0.29 Ea	23.7 ± 1.96 ABa
	+K+D	112.5 ± 10.97 DEb	40 ± 2.46 Dab	20.6 ± 0.99 Fb	20.2 ± 0.72 Db
	–K	133.4 ± 2.62 BCDab	38 ± 2.27 DEb	21.8 ± 0.39 EFa	20.5 ± 0.41 Db
	–K+D	108.8 ± 7.90 DEc	33 ± 3.19 Ec	20.1 ± 1.31 Fb	19.2 ± 0.57 Ec
XZ54	+K	161.6 ± 2.52 Aa	56 ± 3.45 Aa	29.1 ± 0.62 Aa	24.7 ± 1.66 Aa
	+K+D	88.6 ± 8.02 Ec	36 ± 1.52 DEbc	23.6 ± 0.37 DEb	22.9 ± 1.38 BCab
	–K	131 ± 8.44 BCDb	42 ± 0.8 CDb	22.1 ± 0.53 Ebc	21.5 ± 0.93 Cab
	–K+D	89 ± 2.30 Ec	34 ± 2.16 Ec	21.4 ± 0.36 EFc	20.0 ± 1.48 Db

Different capital and small letter(s) indicate significant differences (*p* < 0.05) among the genotypes and among the 4 treatments within each genotype, respectively. +K, +K+D, –K, and K+D, correspond to basic nutrition solution with normal K of 1 mM K (BNS), BNS + 20% Polyethylene glycol 6000 (PEG) induced drought stress, BNS without K (K deficiency), and BNS without K + PEG, respectively.

**Table 2:** Effect of K nutrition on shoot and root Ca, Na, K, Mg concentrations and Na^+^/K^+^ ratio of three barley genotypes in response to drought stress.

Genotype	Treatment	Ca	K	Mg	Na	Na^+^/K^+^ Ratio
		mg g^–1^ DW				
		Shoot mineral concentrations				
XZ5	+K	0.61 ± 0.13 Cb	39.16 ± 0.4 Aa	0.81 ± 0.15 Cb	1.30 ± 0.17 Ea	0.03 Fa
	+K+D	1.30 ± 0.25 Aa	36.83 ± 0.74 ABa	0.79 ± 0.13 Cb	1.23 ± 0.20 Ea	0.03 Fa
	–K	0.74 ± 0.04 Cb	32.17 ± 1.94 BCab	1.39 ± 0.09 Ba	1.58 ± 0.31 DEa	0.05 EFa
	–K+D	1.19 ± 0.17 ABa	28.03 ± 1.06 Cb	1.22 ± 0.23 Ba	1.58 ± 0.05 DEa	0.06 EFa
						
Tadmor	+K	0.87 ± 0.20 BCb	28.86 ± 4.37 Ca	0.76 ± 0.13 CDb	1.3 ± 0.24 Eb	0.05 EFc
	+K+D	1.29 ± 0.15 Aa	21.00 ± 1.57 Fb	0.43 ± 0.02 DEbc	1.72 ± 0.57 DEb	0.08 Ebc
	–K	0.77 ± 0.06 Cb	14.57 ± 1.21 GHc	1.44 ± 0.27 Ba	2.51 ± 0.30 BCa	0.17 Ca
	–K+D	0.77 ± 0.21 Cb	13.46 ± 1.09 Hc	0.28 ± 0.07 Ec	1.65 ± 0.33 DEb	0.12 CDb
						
XZ54	+K	0.80 ± 0.13 Cb	24.52 ± 1.27 Ea	0.59 ± 0.24 CDEbc	1.81 ± 0.39 CDEc	0.07 Ec
	+K+D	1.17 ± 0.27 Aba	19.58 ± 2.72 FGb	0.68 ± 0.31 CDbc	2.10 ± 0.46 BCDbc	0.11 CDc
	–K	0.63 ± 0.04 Cb	15.25 ± 1.43 Gbc	1.84 ± 0.17 Aa	2.55 ± 0.19 Bb	0.17 Cb
	–K+D	0.70 ± 0.11 Cb	12.52 ± 0.97 Hc	0.81 ± 0.06 Cb	4.10 ± 0.25 Aa	0.33 Aa
		Root mineral concentrations				
XZ5	+K	2.40 ± 0.56 BCDab	136.59 ± 17.66 Aa	3.42 ± 0.56 ABa	3.49 ± 0.67 CDb	0.03 Db
	+K+D	1.34 ± 0.13 Db	101.06 ± 24.51 Bb	2.41 ± 0.39 CDb	2.18 ± 0.38 Db	0.02 Db
	–K	2.53 ± 0.18 ABCa	79.00 ± 5.69 CDbc	3.79 ± 0.73 Aa	8.05 ± 0.47 Ba	0.1 Aa
	–K+D	1.87 ± 0.13 Dab	57.49 ± 4.02 DEc	2.29 ± 0.09 Db	3.53 ± 0.62 CDb	0.06 Cab
Tadmor	+K	3.17 ± 0.98 ABab	97.16 ± 21.53 Ba	3.47 ± 1.09 ABa	4.00 ± 1.25 CDb	0.04 CDb
	+K+D	2.41 ± 0.77 BCDab	86.55 ± 7.36 Cb	2.69 ± 0.55 BCDa	5.04 ± 0.96 Cb	0.06 Cb
	–K	3.33 ± 0.58 Aa	75.08 ± 10.45 CDbc	3.60 ± 0.71 ABa	9.83 ± 0.43 ABa	0.13 Aa
	–K+D	2.11 ± 0.79 CDb	47.34 ± 10.16 Ec	2.81 ± 0.67 BCDa	4.62 ± 0.75 Cb	0.1 Aa
XZ54	+K	2.55 ± 0.48 ABCDa	101.02 ± 13.04 Ba	3.37 ± 0.29 ABCab	3.64 ± 0.59 CDb	0.04 CDb
	+K+D	1.78 ± 0.11 Da	82.96 ± 4.96 Cb	2.25 ± 0.13 Dc	3.01 ± 0.62 CDb	0.04 CDb
	–K	2.66 ± 0.09 ABCa	78.71 ± 6.65 CDb	4.02 ± 0.64 Aa	10.62 ± 2.4 Aa	0.13 Aa
	–K+D	1.63 ± 0.19 Da	53.08 ± 4.11 DEc	2.74 ± 0.32 BCDbc	4.15 ± 0.68 CDb	0.08 BCb

Different capital and small letter(s) indicate significant differences (*p* < 0.05) among the genotypes and among the 4 treatments within each genotype, respectively. +K, +K+D, –K, and –K+D, correspond to basic nutrition solution with normal K of 1 mM K (BNS), BNS + 20% Polyethylene glycol 6000 (PEG) induced drought stress, BNS without K (K deficiency), and BNS without K + PEG, respectively.

## Data Availability

All data supporting the conclusions of the present study have been documented in this article.

## Supplementary Materials

The following are available online at https://www.mdpi.com/article/10.3390/ijms222313100/s1.
