# Supplementary material for: Mechanistic Insights into Potassium-Conferred Drought Stress Tolerance in Cultivated and Tibetan Wild Barley: Differential Osmoregulation, Nutrient Retention, Secondary Metabolism and Antioxidative Defense Capacity
Source: Int J Mol Sci. 2021 Dec 3;22(23):13100. doi: 10.3390/ijms222313100 (PMC8658718; doi:10.3390/ijms222313100)
Supplement: Supplementary file 1 [file ijms-22-13100-s001.zip › ijms-1417164-supplementary.pdf]

Supplementary Materials

Table S1. *F*-value/*P*-value for the effect of K nutrition on growth parameters of three barley genotypes in response to drought stress.

Source	*F*-value/ *P-value*			
	**Shoot DW** (μg plant^{-1})	**Root DW** (μg plant^{-1})	**Shoot height** (cm)	**Root length** (cm)
**Genotype**	3.59/0.036*	3.54/0.0376*	10.74/0.01**	9.65/0.0003**
**Treatment**	23.37/0**	3.85/0.0157*	7.85/0.0169*	3.61/0.0205*
**G×T**	2.72/0.025*	5.41/0.0003**	4.2053/0.002**	1.4/0.2464

G×T, Interaction between genotype and treatment; * and **, significant at 0.05 and 0.01 probability level, respectively.

Table S2. *F*-value/*P*-value for the effect of K nutrition on photosynthetic parameters of three barley genotypes in response to drought stress.

Source	*F*-value/ *P-value*					
	**SPAD**	***Fv/Fm***	***Pn*** (μmol [CO_2] $m^{-2} s^{-1}$)	***Gs*** (mol H_2O $m^{-2} s^{-1}$)	***Ci*** (μmol CO_2 mol^{-1})	***Tr*** (mmol H_2O $m^{-2} s^{-1}$)
**Genotype**	19.85/0.002**	4.813/0.0185*	17.14/0.0033**	3.414/0.1023	0.342/0.7232	4.02/0.078
**Treatment**	18.6/0.0019**	32.11/0**	27.736/0.0006**	8.561/0.0138*	13.55/0.0044**	11.91/0.006**
**G×T**	7.03/0**	1.4/0.26	4.27/0.0008**	12.786/0**	8.757/0**	12.5/0**

G×T, Interaction between genotype and treatment; * and **, significant at 0.05 and 0.01 probability level, respectively.

Table S3. *F*-value/*P*-value for the effect of K nutrition on shoot Ca, Na, K, Mg concentrations and Na+/K+ ratio of three barley genotypes in response to drought stress.

Source	*F*-value/*P-value*									
	**Shoot mineral concentrations** (mg g^{-1} DW)					**Root mineral concentrations** (mg g^{-1} DW)				
	**Ca**	**K**	**Mg**	**Na**	**Na^+/K^+ ratio**	**Ca**	**K**	**Mg**	**Na**	**Na^+/K^+ ratio**
**Genotype**	1.65/0.215	121.52/0**	7.82/0.0027 **	26.23/0**	42.15/0**	3.644/0.043*	123.20/0* *	0.253/0 .78	4.994/0. 016*	4.2/0.028*
**Treatment**	13.77/0**	37.3/0.0003**	37.44/0**	10.44/0.000 2**	30.1/0**	9.64/0.0003**	37.79/0**	11.4/0. 0001**	49.61/0 **	17.76/0**
**G×T**	1.933/0.120 2	1.0138/0.44	4.8/0.002 9**	6.00/0.00 08**	7.6/0.000 2**	0.206/0.97	1.13/0.4	0.441/ 0.843	1.15/0. 367	0.33/0.914

G×T, Interaction between genotype and treatment; * and **, significant at 0.05 and 0.01 probability level, respectively.

Table S4. *F*-value/*P*-value for the effect of K nutrition on shoot and root antioxidant enzyme activities and MDA contents of three barley genotypes in response to drought stress.

Source	*F*-value/*P-value*				
	**SOD** (Unit mg^{-1} protein)	**POD** (Unit mg^{-1} protein)	**APX** (Unit mg^{-1} protein)	**CAT** (Unit mg^{-1} protein)	**MDA** (nmol g^{-1} FW)
**Shoot**					
**Genotype**	9.55/0.001**	2.23/0.131	10.20/0.0007**	2.69/0.09	14.54/0.005**
**Treatment**	15.62/0**	5.53/0.005**	29.33/0**	14.86/0**	39.07/0.0002**
**G×T**	4.24/0.0055**	1.095/0.396	5.38/0.001**	4.92/0.002**	4.126/0.0063**
**Root**					
**Genotype**	1.71/0.26	2.697/0.146	16.21/0**	2.2/0.14	12.55/0.0072**
**Treatment**	3.66/0.083	1.2662/0.37	21.43/0**	6.15/0.003**	8.91/0.0125*
**G×T**	16.15/0**	24.471/0**	7.04/0.0003**	0.976/0.47	7.82/0.0001**

G×T, Interaction between genotype and treatment; * and **, significant at 0.05 and 0.01 probability level, respectively.

Table S5. *F*-value/*P*-value for the effect of K nutrition on shoot and root H+/K+ ATPase and Ca^{2}+/Mg^{2}+ ATPase activities of three barley genotypes in response to drought stress.

Source	*F*-value/ *P-value*			
	**Shoot H^+/K^+ ATPase** (μmol Pi mg^{-1} protein h^{-1})	**Root H^+/K^+ ATPase** (μmol Pi mg^{-1} protein h^{-1})	**Shoot Ca^{2+}/Mg^{2+} ATPase** (μmol Pi mg^{-1} protein h^{-1})	**Root Ca^{2+}/Mg^{2+} ATPase** (μmol Pi mg^{-1} protein h^{-1})
**Genotype**	50.1455/0**	74.6149/0**	3.7012/0.0412*	2.1047/0.1458
**Treatment**	27.3965/0**	91.6315/0**	7.0094/0.0018**	4.4523/0.0137*
**G×T**	11.1092/0**	43.2296/0**	3.1811/0.0211*	1.2271/0.3303

G×T, Interaction between genotype and treatment; * and **, significant at 0.05 and 0.01 probability level, respectively.

Table S6. *F*-value/*P*-value for the effect of K nutrition on shoot and root proline, glycine betaine, soluble sugar and soluble protein contents of three barley genotypes in response to drought stress.

Source	*F*-value/*P-value*							
	Shoot				Root			
	**Proline** (μg g^{-1} FW)	**Glycine betaine** (μg g^{-1} DW)	**Soluble sugars** (mg g^{-1} DW)	**Soluble proteins** (μg g^{-1} FW)	**Proline** (μg g^{-1} FW)	**Glycine betaine** (μg g^{-1} DW)	**Soluble sugars** (mg g^{-1} DW)	**Soluble proteins** (mg g^{-1} DW)
**Genotype**	91.31/0**	57.52/0**	6.144/0.0076**	1610.10/0**	0.81/0.4575	13.42/0.0002**	271.503/0**	808.56/0**
**Treatment**	546.43/0**	186.84/0**	133.898/0**	694.5/0**	2.87/0.0596	42.97/0**	140.34/0**	16.44/0**
**G×T**	22.88/0**	24.91/0**	128.969/0**	534.87/0**	6.91/0.0003**	1.77/0.151	109.66/0**	39.32/0**

G×T, Interaction between genotype and treatment; * and **, significant at 0.05 and 0.01 probability level, respectively.

Table S7. *F*-value/*P*-value for the effect of K nutrition on secondary metabolism related enzymes of three barley genotypes in response to drought stress.

Source	*F*-value/*P-value*							
	Shoot				Root			
	**PAL** (Unit mg^{-1} protein)	**PPO** (Unit mg^{-1} protein)	**CAD** (μmol mg^{-1} protein)	**SKDH** (μmol mg^{-1} protein)	**PAL** (Unit mg^{-1} protein)	**PPO** (Unit mg^{-1} protein)	**CAD** (μmol mg^{-1} protein)	**SKDH** (μmol mg^{-1} protein)
**Genotype**	0.27/0.77	18.04/0**	0.8303/0.4491	17.04/0**	14.49/0.0001**	19.2/0**	34.8/0**	4.96/0.0167*
**Treatment**	62.94/0**	4.72/0.011*	33.4/0**	26.9/0**	16.44/0**	6.37/0.0029**	12.19/0.0001**	0.94/0.4401
**G×T**	18.72/0**	1.69/0.171	4.71/0.0032**	6.1/0.0007**	11.87/0**	3.0454/0.0253*	59.63/0**	4.16/0.0061**

G×T, Interaction between genotype and treatment; * and **, significant at 0.05 and 0.01 probability level, respectively.

Table S8. *F*-value/*P*-value for the effect of K nutrition on secondary metabolites of three barley genotypes in response to drought stress.

Source	*F*-value/*P-value*							
	**Shoot**				**Root**			
	**Callose** (μg g^{-1} FW)	**Phenols** (mg g^{-1} FW)	**Flavonoids** (mg g^{-1} FW)	**Chitinase** (U mg^{-1} protein)	**Callose** (μg g^{-1} FW)	**Phenols** (mg g^{-1} FW)	**Flavonoids** (mg g^{-1} FW)	**Chitinase** (U mg^{-1} protein)
**Genotype**	8.8/0.0164*	5.09/0.051	1.996/0.217	4.35/0.068	7.04/0.0267	0.81/0.488	1.155/0.377	4.27/0.0701
**Treatment**	3.7/0.0812	0.86/0.513	34.7/0.0003**	4.505/0.056	47.9/0.0001**	19.5/0.0017**	0.44/0.731	5.17/0.042*
**G×T**	50.21/0**	2853.02/0**	689.12/0**	37.4/0**	28.006/0**	110.09/0**	46.57/0**	165.59/0**

G×T, Interaction between genotype and treatment; * and **, significant at 0.05 and 0.01 probability level, respectively.

Table S9. *F*-value/*P*-value for the effect of K nutrition on antioxidant enzymes related gene expression of three barley genotypes in response to drought stress.

Source	*F*-value/*P-value*							
	**Shoot**				**Root**			
	CAT1	***CAT2***	***CuZn-SOD***	***APX***	***CAT1***	***CAT2***	***CuZn-SOD***	***APX***
**Genotype**	0.047/0.95	2.73/0.143	11.24/0.0004**	4.34/0.068	1.16/0.37	5.45/0.044*	4.964/0.053	7.93/0.02*
**Treatment**	2.22/0.19	1.21/0.38	10.33/0.0002**	8.30/0.014*	2.5/0.16	6.32/0.028*	2.98/0.11	2.78/0.133
**G×T**	55.99/0**	103.77/0**	9.14/0**	11.52/0**	19.7/0**	68.71/0**	27.71/0**	14.33/0**

G×T, Interaction between genotype and treatment; * and **, significant at 0.05 and 0.01 probability level, respectively.

Table S10. *F*-value/*P*-value for the effect of K nutrition on secondary metabolism related gene expression of three barley genotypes in response to drought stress.

Source	*F*-value/*P-value*							
	**Shoot**				**Root**			
	PAL	***PPO***	***CAD***	***SKDH***	***PAL***	***PPO***	***CAD***	***SKDH***
**Genotype**	1.017/0.42	0.63/0.565	0.273/0.77	2.6/0.154	4.74/0.058	1.332/0.33	6.12/0.036*	4.1/0.076
**Treatment**	0.79/0.54	1.41/0.33	0.75/0.562	6.02/0.03*	1.96/0.22	1.89/0.23	1.663/0.273	2.5/0.16
**G×T**	30.89/0**	448.75/0**	352.3/0**	33.75/0**	30.98/0**	1046.8/0**	102.48/0**	36.13/0**

G×T, Interaction between genotype and treatment; * and **, significant at 0.05 and 0.01 probability level, respectively.

Table S11. Candidate genes and corresponding primers used for qRT-PCR experiments.

Gene	Forward primer (5'-3')	Reverse primer (5'-3')
HvCZSOD	CACCCAGGAGGGAGATGGTA	GGTTGAAGTGCGGTCCTACA
HvAPX1	CGGAGCTTTTGAGTGGTGACA	CCGCAGCATATTTCTCCACAA
HvCAT1	CCCGTCTGGAACAACAAC	CCCCGTGCATGAACAAC
HvCAT2	CGACGACAAGATGCTGCAGT	TGGTTGTTCTTGAAGCCGC
PAL	CCCAAGTTTGGACTATGGCT	ATCTTGGTTGTGCTGCTCTG
PPO	TCTACAACGAGAGGCGTGAC	GGTCGATCAGCTGGTCTCTT
CAD	AAGAGACAGGAGGCTTTGGA	GGTAAGGCTCAAGAGGGTGA
SKDH	TCTTGCAAACACAACAGCAA	CACATTCTGCAGCTTCCCTA

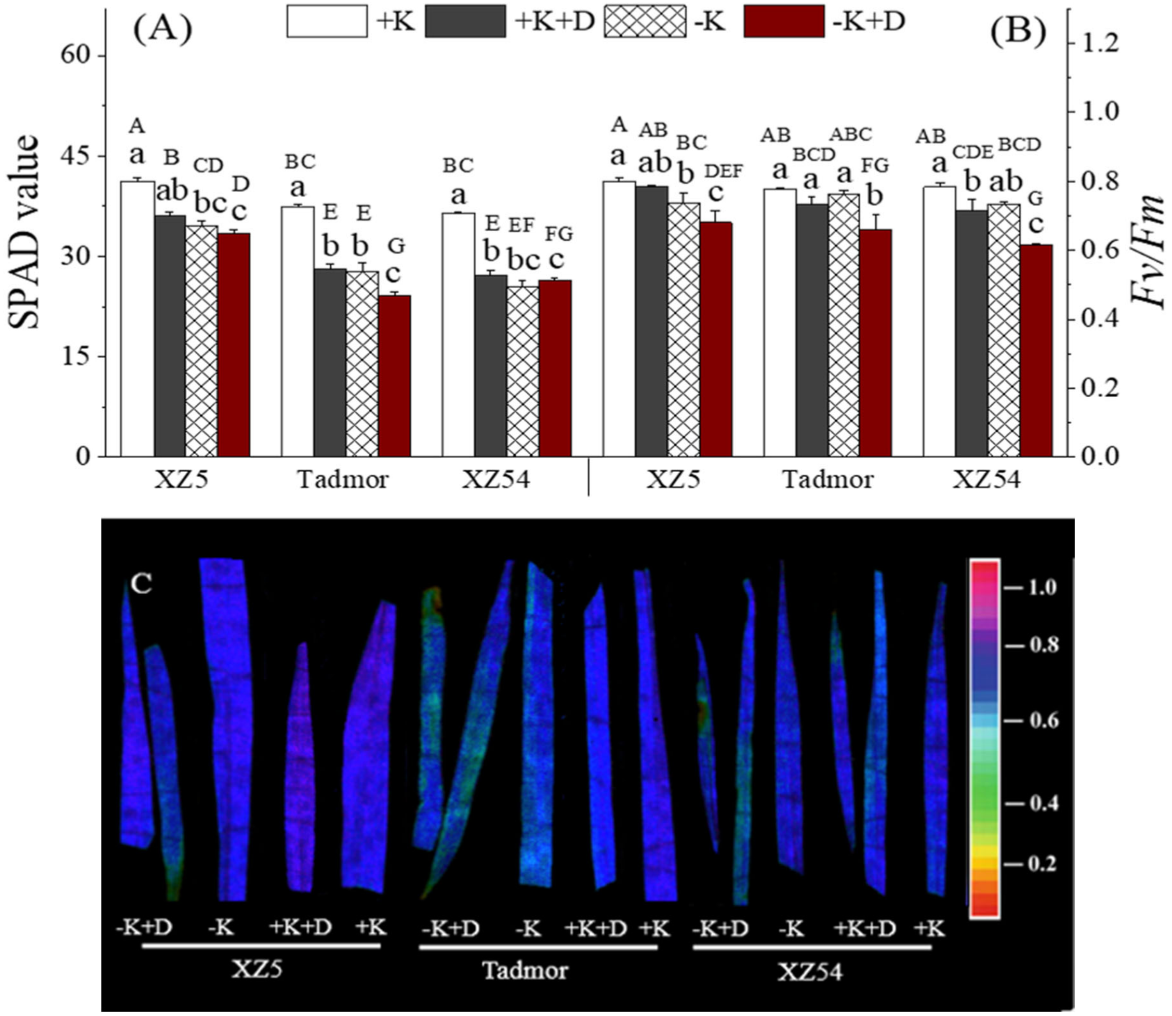

Figure S1. Effect of drought and K deprivation, applied either alone or in combined, on relative chlorophyll contents (SPAD values) (A) and chlorophyll fluorescence yield (Fv/Fm) (B), and false color images of Fv/Fm in response to drought stress (C) of three barley genotypes. Different capital and small letter(s) indicate significant differences ($P<0.05$) among the genotypes and among the 4 treatments within each genotype, respectively. Error bars represent standard deviations ($n = 4$).

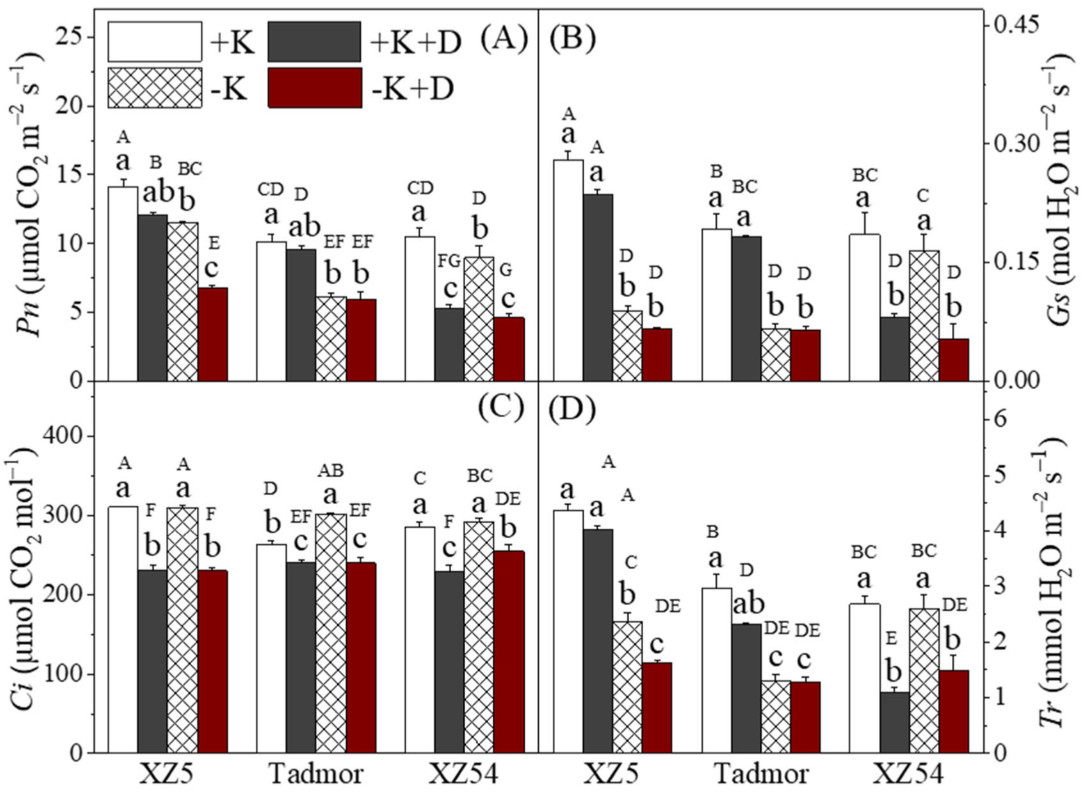

Figure S2. Effect of drought and K deprivation, applied either alone or in combined, on photosynthetic parameters of three barley genotypes. Different capital and small letter(s) indicate significant differences ($P<0.05$) among the genotypes and among the 4 treatments within each genotype, respectively. Values in parenthesis express the percentage decrease (-) with respect to control. Error bars represent standard deviations ($n = 3$).

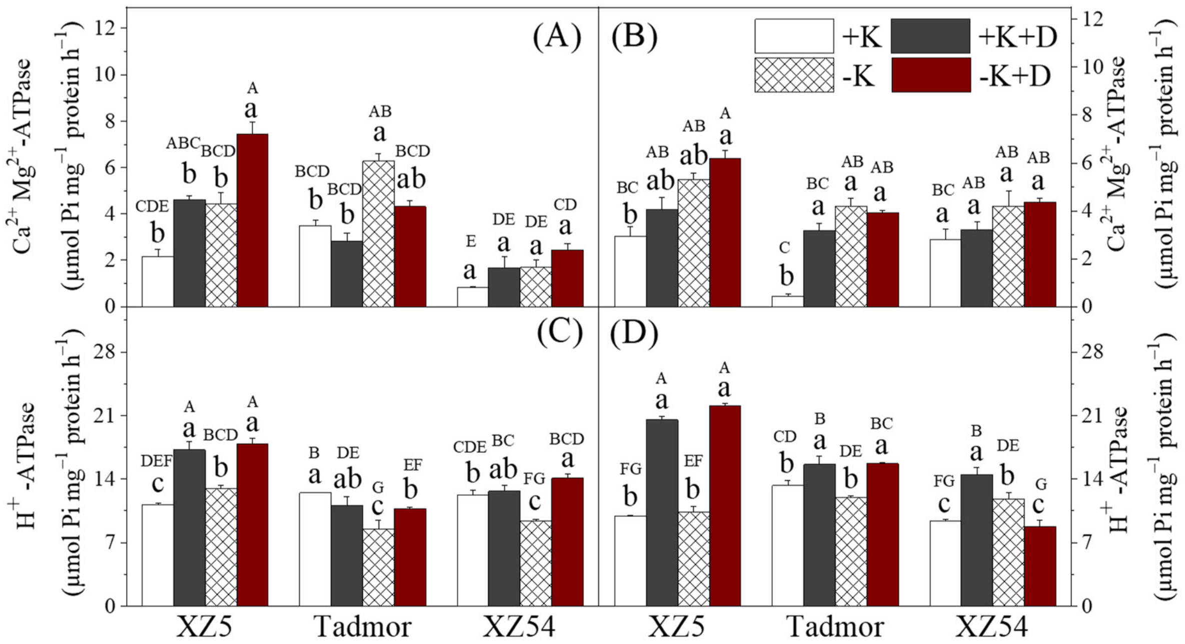

Figure S3. Effect of alone and combined stress of drought and K deprivation on Ca2+ Mg2+-ATPase and H+- ATPase activities in leaves (A, C) and roots (B, D) of three barley genotypes. Different capital and small letter(s) indicate significant differences (P<0.05) among the geno-types and among the treatments within each genotype, respectively. +K (control), +K+PEG, -K, and -K+PEG, correspond to basic nutrition solution with normal K of 1 mM K (BNS), BNS+20% Polyethylene glycol 6000 (PEG), BNS without K (K deprivation), and BNS without K +20% PEG, respectively. Error bars represent standard deviations (n = 3).
